# Integrating Biosystem Models Using Waveform Relaxation

**DOI:** 10.1155/2008/308623

**Published:** 2008-11-10

**Authors:** Linzhong Li, RobertM Seymour, Stephen Baigent

**Affiliations:** 1 Institute for Energy Technology (IFE), P.O. Box 40, 2027 Kjeller, Norway; 2 Department of Mathematics, University College London, Gower Street, London WC1E 6BT, UK; 3 Center for Mathematics and Physics in the Life Sciences and Experimental Biology (CoMPLEX), University College London, Wolfson House, 4 Stephenson Way, London NW1 2HE, UK

## Abstract

Modelling in systems biology often involves the integration of component models into larger composite models. How to do this systematically and efficiently is a significant challenge: coupling of components can be unidirectional or bidirectional, and of variable strengths. We adapt the waveform relaxation (WR) method for parallel computation of ODEs as a general methodology for computing systems of linked submodels. Four test cases are presented: (i) a cascade of unidirectionally and bidirectionally coupled harmonic oscillators, (ii) deterministic and stochastic simulations of calcium oscillations, (iii) single cell calcium oscillations showing complex behaviour such as periodic and chaotic bursting, and (iv) a multicellular calcium model for a cell plate of hepatocytes. We conclude that WR provides a flexible means to deal with multitime-scale computation and model heterogeneity. Global solutions over time can be captured independently of the solution techniques for the individual components, which may be distributed in different computing environments.

## 1. Introduction

A component-based methodology is explicitly or implicitly widely applied to the understanding and modelling of biological systems. For example, to represent a cell and its wide range of functions, we have to integrate individual models for relevant metabolic, signalling, and gene expression pathways, as well as the associated biophysical processes for intracellular, intercellular and extracellular transport. At the next scale up, a tissue or organism level model requires the integration of different kinds of cell function and cell-cell communication in their intra-and extracellular environments. This is typical of the bottom-up approach to systems biology, in contrast to the top-down approach, which tends to start from the system as a whole (see [1] for a thorough discussion of such general issues).

Living systems are maintained by a continuous flow of matter and energy, and thus any biological system inevitably will be a subsystem of a larger one. Therefore, the biological modeller typically has to deal with an open, multilevel and multicomponent system, the perceived nature of which evolves with our increasing understanding. A key feature of such a system is the interactions (or coupling in mathematical terminology) among its heterogeneous components and with the external environment, in which a variety of spatial and temporal scales may exist. These interactions may be strong or weak, unidirectional or multidirectional, depending on the current state of the system, and often generate emergent properties through nonlinear interactions. The diversity of existing modelling techniques adds a further layer of complexity to this situation. Thus models of individual components can be based on different modelling formalisms, such as differential equations, discrete time or discrete event simulations, different levels of abstraction of system behaviours, the extent of available knowledge, and the nature of the phenomena being studied.

It can take many years and enormous effort by many researchers across disciplines to build up a model of a complex biological system, and this only on a coarse-grained level consistent with current understanding, which therefore is constantly in need of refinement as techniques and understanding improve. A general issue, therefore, naturally arises: how do we systematically integrate both existing and well-established, as well as new, or more refined versions of old, model components in order to build up a larger model system with minimal modification of the internal structure of component submodels?

When describing the behaviour of a complex model system, traditionally we tend to view the system as a whole, implying that the coupling between component parts is implicitly represented. This is driven, in part, by the need to specify suitable mathematical spaces in which whole-system solutions should lie. However, from a computational point of view, it is unnecessary to solve a system as a whole. In contrast to this traditional approach, it is often more natural to construct whole-system behaviours by solving individual components separately, and then to consider the coupling explicitly. This is also often more consistent with developing understanding of the system through the study of separate, isolated components, and makes it possible to update model components individually as knowledge of the detailed biology evolves. Moreover, this approach provides a framework for integrating heterogeneous models (as components of a larger system), which can be distributed in different computational environments.

In the context of integrating biological models, a computational framework under a multicomponent system speci cation (see [2] for a formal definition) should possess the following features.

(i) It must be able to represent biological scales both faithfully and economically. This requires a multiscale algorithm, which aims not only to capture the individual biological scales associated with each component but also to resolve the differences of scale between components in a computationally efficient way.

(ii) The framework should provide the flexibility for integrating models based on different mathematical formalisms, such as deterministic and stochastic simulation. Different mathematical formalisms are often forced upon us by the existence of different spatial or temporal scales.

(iii) The framework should support encapsulated components. When a new model for a component is developed, we should be able to include it easily, without changing the rest of the framework. This requirement can be termed "plugability", for example [3].

(iv) It should also support linking components represented in different software environments, so as to allow new models to be constructed from existing models with minimal changes.

These basic requirements call for a general framework based on a combination of modular, object-oriented design and agent-oriented design. Modular and object-oriented design provide the flexibility for plugability, and agent-oriented design facilitates the interoperability of coupled models [4]. However, all these designs should be based on a solid theoretical foundation, and also provide a practical means to capture the global dynamic solutions independently of the solution techniques that might be employed for individual components. Suggesting such a methodology is the aim of this work.

Work towards these aims has been published in [3], where a novel algorithm based on a discrete event scheduler was presented in an attempt to meet some of the above requirements. Although successful in many respects, this algorithm is unable to achieve multitime-scale computation for a system with bidirectionally coupled components because of the excessive computational cost of very frequent communication between components. However, bidirectional coupling is common in biological systems due to the ubiquity of feedback mechanisms. For example, in cell signalling, signals are tightly regulated with positive and negative feedbacks that are bidirectional, with commands travelling both from outside-in and inside-out [5].

Here, we offer an approach to deal with bidirectionally coupled components that meets the requirements listed above: waveform relaxation (WR), a flexible numerical method for computing solutions to a system of ordinary differential equations (ODEs) that enables the integration of independently treated subsystems, by using outputs of subsystems as inputs to others and vice versa [6]. When the idea behind the WR method is generalised to a wide range of modelling techniques, it supports a multiscale algorithm that can integrate models of different mathematical forms and provides a way to characterise the dynamical solutions over time and space, independently of the solution technique that might be employed for individual components. However, here we restrict attention to ODE models.

In Section 2, we present a general formulation of the problem of model composition from component models in the context of ODE systems, and in Section 3, the WR method is briefly introduced and generalised, accompanied by some general discussion of how the method is implemented, especially when employed for a discrete event strategy. In Section 4, four case studies are presented to show the capability of the method to cope with different aspects of simulating biological systems. Finally, in Section 5 the results are summarised and discussed.

## 2. Model Formulation

For simplicity, we consider ODE models to describe briefly our model formulation.

Suppose a given whole-system ODE model  describing a complex biological system. We assume that the state space is an open subset  of an -dimensional Euclidean space , and that the model takes the form of a nonautonomous ODE system:(1)

where  satisfies some suitable Lipschitz condition to ensure existence and uniqueness of solutions for  . We assume that  may be decomposed into  component subsystems , , in the following sense.  is specified by a state space  of dimension , together with a linear embedding  which expresses state variables for the component  as state variables for the composite system . The set of component systems  then forms a *decomposition* of the system  provided that every state variable of  can be expressed as a sum of state variables of component subsystems. More formally, if  is a state vector for the whole system, then we can find state vectors  for , such that  (this expression need not be unique). This formulation allows the possibility that different components can share some of the same state variables, a device that can facilitate more efficient computation. In what follows, we drop explicit reference to the embeddings  to keep the notation simple.

Using  as a component state vector, and  for the whole-system state, we obtain a decomposed model of the form(2)

Now consider the inverse problem of integrating ODE components  into a single, composite system . In this case, we have to supply the functions that define the way in which the components are coupled together.

First, consider an individual (i.e., uncoupled or isolated) ODE component. Let ,  denote subsets of finite-dimensional Euclidean spaces, and let  be a locally Lipschitz function. Then, the th ODE component is assumed to be specified in the form(3)

Here,  is the *internal state vector* belonging to the state space , whose elements are the *internal state variables* associated to the th component. The second component, , is a supplied time-dependent input that will be used to communicate intercomponent interactions and to represent parameter values, such as internal parameters and external forcing functions (i.e., a vector of *control variables* in the language of control theory). For a completely isolated, autonomous component, the  would be just the parameter values needed to run the component alone. However, when not isolated, say as part of an integrated system consisting of  components, the vector function  can be further decomposed into two parts, , one being the *external state vector*, whose elements (the *external state variables* of component ) are internal state variables belonging to other components via the intercomponent coupling, and the other being a vector , representing other internal and external parameters and controls. Thus we assign two types of state variable to each component: internal state variables and external state variables, with the internal state variables being always state variables of the component independent of whether the component is isolated or not, while the external state variables become state variables (of some other subsystem) only when these components are combined to form a composite system.

For example, in an isolated metabolic system without protein synthesis and degradation, the parameters  are the concentrations and the kinetic and binding constants of the enzymes involved, as well as the concentration of external substrates, which are determined by external conditions but not controlled by the system, and can be time dependent. The internal state variables are then the time-dependent concentrations of the intermediary metabolites. When the system is combined with relevant signalling and gene regulatory pathways, parts of the parameters , say the concentrations of the enzymes in the metabolic system, actually become the state variables (i.e., internal state variables) of the signalling and gene regulatory pathways. Thus when we are considering behaviours of the whole system, some parameters originally attributed to these subsystems should not be treated as parameters of the whole system. However, these biologically distinguishable pathways have their disparate time scales, and a variable in one subsystem with a low-relaxation time may be viewed as a bifurcation parameter for the others when the subsystems are isolated.

For such a composed system, once , , are specified, a dependence digraph can be constructed, which represents the connectivity of the network, and the bidirectional coupling between components can be rigorously defined in terms of graph theory. Here, for the purpose of representing the WR method in its generic form, we define instead an *influence set* for each *i*. That is, if  indexes the component subsystems, then  is a set of (external) component indices that influence component . Thus  is the collection of indices of those components, some internal state variables of which are the external state variables of component .

In terms of this conceptualisation, and in order to allow for additional flux transfer between the subsystems modelled by components, we assume that the th component function  in (3) is specified in the form(4)

where the function  is reserved for representing flux transfer with other components. It can be constructed from mass action or some other representation, such as Hill functions, of the fluxes due to the interactions of linked components that contribute to the overall flux balance in the whole system. For example, when we write reaction equations using mass action, introducing a new molecular species will result in new flux terms to the original equations, that is, the second term in (4) will appear, while the first term will keep the same meaning as in the original, isolated system.

A composition of the components  into a single system  results from a specification of the control variables in  and the flux transfer function . Once these are given, the composed system provides an appropriate set of (time-dependent) functions  (). The velocity of  is then given by the functional composition of the supplied component functions  and the interaction functions . The resulting composed system  is therefore described by the dynamical system (4).

The above formulation suggests that a generic form for any submodel should be provided with the form (4) with a dummy flux term. As an isolated system, this term is set to zero, but as a component in an integrated model, this term can be formed according to flux contributions from the interactions of linked components. The advantage of this formulation is to provide the flexibility to link to other potential models without altering the internal structure of the original model when the WR method, which will be introduced in Section 3, is applied.

## 3. Computational Approach

In this section, we first provide a brief introduction to the waveform relaxation method (WR), originally developed for the parallel computation of ODEs [6]. We formally generalise the method and discuss some issues relevant to its implementation and efficient computation.

### 3.1. The Waveform Relaxation Method

We illustrate the WR method using a system decomposed into communicating components. Thus suppose we have a system described by a set of differential equations, decomposed into two subsystems (components) 1 and 2 of the form(5)

where  and  are the state vectors for components 1 and 2, respectively. Then we have two iterative implementation schemes for WR as follows.

(1) *Jacobi Wr Method*(6)

for .

(2) *Gauss-Seidel Wr Method*(7)

for .

Roughly speaking, the Jacobi WR method updates a component based upon the states of all components in the previous iteration, while in the Gauss-Seidel WR method, the new state may also depend on the newly updated states of the current iteration, in addition to states from the previous iteration. The Jacobi and Gauss-Seidel methodologies are widely employed in numerical computation, such as for solving linear or nonlinear algebraic equations and finite difference equations in addition to ODEs. A classical cellular automaton simulation is an example of the Jacobi WR method.

A very important message obtained from the WR method is that a large system can be split into small components, which can be computed independently, while the coupling between components can be realised by an iterative procedure. Conversely, components can be computed independently and by specifying the interfaces between the components and performing an iterative procedure, we are actually simulating a larger system formed by these components. Therefore, we interpret the WR method as an iterative procedure to represent bidirectionally coupled components in such a way that each component can be calculated independently. Of course, if components are unidirectionally coupled, then there is no need to do the iteration and a sequential calculation is sufficient.

Following the notation of Section 2, we can write the WR method in its generic form. For the Jacobi WR method, we have(8)

for , , with  and .

In this iterative procedure, the external state variables take their values from the previous iteration and determine the internal state variables in the current iterate. Thus the iteration loop itself does not play a role, since it does not take account of the specific topological structure of the network. Of course, initial guesses for , , have to be given in order to start the iteration.

Much of the complexity of the iterative procedure comes from the Gauss-Seidel WR method, and it should be defined in terms of the dependence digraph to take account of the topological structure of the network. For simplicity, for an -component system, we assume an iterative loop has been predetermined, say, in the sequential order of  (by relabelling components if necessary), with influence set for the th component , where . The method can be formally defined by(9)

, , where(10)

These two methods are examples of continuous waveform relaxation (continuous referring to the time variable). In a numerical implementation of the method, each set of continuous differential equations has to be discretised into a set of difference equations, and this results in a discrete waveform relaxation. Under fairly general conditions, both continuous and discrete WRs are convergent to the theoretical solutions [6]. Thus we have

for any set of initial conditions , . Moreover, the limit functions  satisfy the original system (1). The rate of convergence of the iterates depends on the length  of the time interval on which the iteration is performed, and the way the system is partitioned into subsystems.

Splitting a large system into smaller components has another advantage other than the obvious one for parallel computation. Fast and slow varying components may exist, and these can be solved economically by integrating the slow components using larger step sizes than the fast components, with adaptive step size methods employed to realise this. Otherwise, if we solve the system as a whole, integration must be performed with a single time step, which will be determined by the fastest time scale among all the components.

Although here we present the WR method for a system specified by ODEs, the methodology is applicable to many kinds of system specification. In particular, WR has been generalised to stochastic differential equations (SDEs) [7]. The most widely applied numerical method for simulating stochastic systems is the famous Gillespie method [8]. This method can also be interpreted in terms of WR using Gibson and Bruck's formulation [9]. Specifically, we can treat each reaction as a component of the system of interest, and the iteration procedure in WR can be interpreted as updating states following the dependence graph, since this is a discrete event simulation and there are no simultaneous events; that is, there are no bidirectionally-coupled events. However, such an interpretation is not practically useful without further exploration of efficiency issues.

A more practical application of the WR method lies in simulation for hybrid models, which combine stochastic descriptions (say, for gene expression) and deterministic descriptions (say, for signal transduction). The requirement for such model heterogeneity is common in modelling biological systems [10,11] and is a significant challenge. In fact, the dynamical systems theory for such hybrid models has been identified as an important future research direction [12]. For such hybrid simulation, the WR method can be applied in a straightforward manner so long as interfaces between deterministic and stochastic submodels can be clearly defined, allowing the WR iteration procedure to be applied to simulate the bidirectional coupling between submodels. In the case studies to be developed in Section 4 below, we will provide examples of this kind of simulation.

The key idea of the WR method is to provide a way to compose a system from its components, or to decompose a system into components, by an explicit specification of the coupling relations among components, independent of the internal specification of these components. The explicit specification of the coupling facilitates an iterative procedure that in one iteration sweeps over the components, updating them based upon the states of other components. In this way, the states of individual components can be computed independently, and their bidirectional communication with states of other components is achieved by this iterative procedure.

Moreover, WR provides a means to compute global solutions over time independently of the solution techniques that might be employed for individual components. Such independence also implies that any multiscale method can be applied to solve components that involve a variety of spatial, as well as temporal scales. Another implication from the WR method is that two different models can be linked together by specifying an interface between them, even without significantly modifying the components (e.g., by adding new flux contributions to the system as in equation (4)) or the solution techniques. That is, WR facilitates model *encapsulation*. This is because communication between components in the implementation of the WR method is carried out by data input and output. Thus when adaptive grid methods both in time and space, stiff solvers, and multirate methods [13] are suitably chosen for all components, a wide range of multiscale computation becomes possible.

### 3.2. Practical Implementation of WR

In the implementation of WR, the time interval  is partitioned into a set of *L* subintervals . The iteration for bidirectionally coupled components is performed on each subinterval sequentially, that is, starting from  and moving to the next after the convergence of the iteration on the current interval is achieved, and so on. This is called a "windowing technique" and its application is necessary to avoid the requirement for excessive storage as well as to reduce the number of iterations. In principle, the subintervals can be chosen adaptively, say representing the largest time scale among all the components in order to maximise the efficiency of a multiscale computation, but here, for illustrative purposes, we suppose that each subinterval is of equal length.

A numerical method is chosen for each component; there is no requirement for the same method to be applied to all components. An interpolation method is also required to facilitate the communication between components through external state variables, which are the inputs to the component under execution. The reason for such a requirement is that when an adaptive method is applied to each component on each iteration, the input values from the external state variables associated to a given component are normally not available. This is because the resulting grid points are not the same for each component and each iteration, and therefore have to be obtained by interpolation for a given component.

As observed earlier, the abstract formulation of a system decomposition in Section 2 allows the possibility of overlapping components; that is, different components sharing some of the same internal state variables. This can potentially result in better convergence properties of the waveform iterates by ameliorating the effects of strong coupling between components [14].

While there is considerable flexibility in the choice of numerical methods for solving individual components and interpolating the solution output, some general rules should be followed in order to obtain high accuracy and efficiency. First, an adaptive step size method should be used to capture the right time scale of each component, and this is where the multitime scale efficiency of WR lies. If models for some components are stiff, then stiff solvers should be used. Second, both the orders of accuracy of integration methods for different components, and the orders of accuracy of the interpolation methods should be consistent so that the accuracy of the whole computation is not lost. In our implementation, we employ both Gear's stiff solver and Prince-Dormand's embedding explicit Runge-Kutta 5(4) [15], and three-point Hermite polynomial interpolation for the differential equation specified system, while Gillespie's method is employed for the stochastic simulation with linear interpolation.

### 3.3. Monitoring and Utilising Varying Coupling Strengths

Coupling among components is a dynamical property of the system, in the sense that two components bidirectionally coupled together by the specification of a network structure does not mean that the two components are strongly coupled for all time. For example, in the simulation of very large-scale integrated (VLSI) circuits, it was found that strong coupling between components only occurs over short-time intervals [16].

Updating all components even when the coupling between some components is found to be weak would be computationally inefficient. Thus we apply the strategy of discrete event simulation to the WR iteration loop [2]. Specifically, in each iteration loop, before executing any component, we examine the variation between the previous iterate and the present one of both internal and external state variables for that component. If the change of these variables is sufficiently small relative to the value of present iterate, then we skip the calculation of the component. The multitime-scale efficiency of a WR algorithm will be dependent on the computational cost for the iteration, and essentially dependent on the coupling strength among the components. The stronger the coupling, the more iterations are needed. On the other hand, the coupling strength of components is dynamically changeable, and therefore the discrete event strategy proposed actually allows us dynamically to follow the change by adaptively reducing or increasing the number of iterations for each component. In the case studies given below, we will demonstrate this by an example. Further issues related to the application of the WR method will be discussed along with each case study.

## 4. Case Studies

We now demonstrate the application of the WR method with four models. The first is a simple cascade of harmonic oscillators discussed in [3]. Although the model is not inspired by a biological system, it serves well as a simple example of the improvement the WR method offers over a standard integration algorithm (i.e., one that does not rely on explicit decomposition and coupling of the model). This model is then modified to include feedback from faster components to slower components giving a bidirectionally coupled system. The second model is based on Höfer's calcium oscillation model [17], reformulated so that a combination of deterministic and stochastic simulation can be executed within the WR framework. The third model is a single-cell calcium model [18] that generates different oscillation patterns ranging from simple periodic oscillations to periodic and chaotic bursting in response to agonist stimulation. The model also has a natural decomposition into 2 modules with distinct time scales. This model therefore provides a suitable test case for validating the convergence property of WR, and the benefits of model decomposition based on time scale differences. Finally, the fourth model, which is also based on Höfer's calcium oscillation model, is a genuinely multicellular model that deals with the synchronisation of calcium oscillations within a plate of heterogeneously coupled hepatocytes.

### 4.1. A Cascade of Harmonic Oscillators

**Figure 1:**
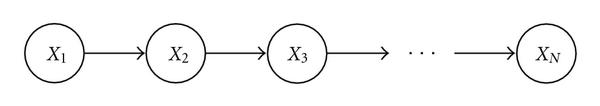
**A cascade of harmonic oscillators with unidirectional coupling**.  with .

A system of  unidirectionally, linearly coupled harmonic oscillators is defined by the following equations:(11)

where the frequency of the th harmonic oscillator is . See Figure 1.

The time scale difference among components is explicit in this model. For instance, the total time scale difference across the frequencies  is about 6 orders of magnitude for .

The application of the Gauss-Seidel WR to this unidirectional model results in a sequential execution of each component, and no iteration loop is necessary. This is because the system has triangular structure owing to the unidirectional coupling, and the solution can be obtained sequentially by solving for the *i*th oscillator as driven by the ()th oscillator.

**Figure 2:**
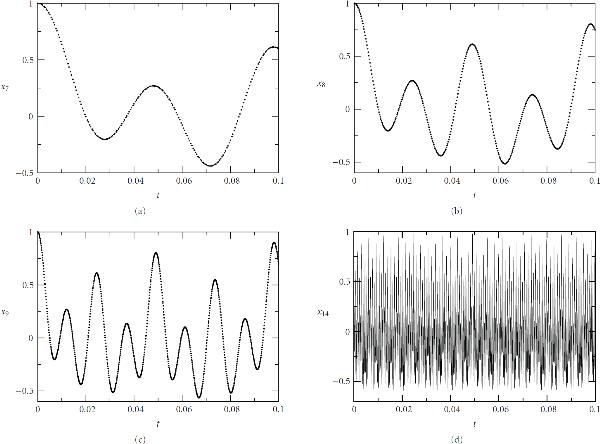
**Computed amplitudes of units 7, 8, 9, 14 in a cascade of  harmonic oscillators with unidirectional coupling with the initial conditions: , **.

The efficiency of this multitime scale computation can be easily understood. We consider the solution on a fixed time interval . Using a WR method based on an adaptive grid method, which precisely follows time-scale variations of component solutions, the number of time steps taken to cover this interval for the th component is proportional to the frequency . Thus the total number of time steps used for all components is proportional to  for large . On the other hand, when a standard adaptive grid method is applied to the whole system, the number of time steps used to reach the end of the interval will be determined by the fastest component (component  in this example), to resolve the variation for this component, regardless of slower variations in other components. That is, the total number of time steps used is proportional to . Thus the WR method is approximately  times faster than a standard flat algorithm. In particular, for , the WR method is approximately 10 times faster. This comparison is based on the cost of evaluation of the right-hand functions of the system, with the computational cost of interpolation, which is fixed for a particular interpolation method, neglected. Figure 2 shows the calculated solutions for oscillators 7, 8, 9, 14.

**Figure 3:**
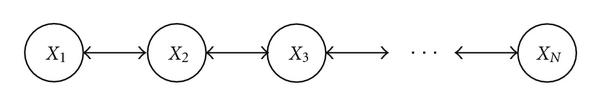
**The harmonic oscillators with bidirectional coupling**.  with , .

This simple picture changes when we modify the model by adding feedbacks from faster components to their neighbouring slower components (Figure 3), thereby rendering the coupling bidirectional. The ability to cope efficiently with such bidirectional feedback is an important property of any numerical methodology, since feedbacks are important features of biological control systems. This system has no specific biological interpretation. Nevertheless, like the unidirectional system considered above, it provides a significant test case for the WR methodology.

The modified system is given by(12)

**Figure 4:**
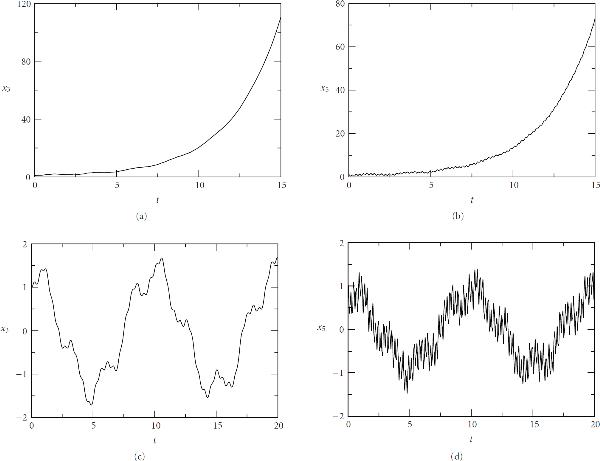
**The harmonic oscillators with bidirectional coupling**. . (a) and (b): , unstable; (c) and (d): , stable. . The initial conditions: , .

The parameter  measures feedback strength. Stability requirements constrain this parameter. For real , the stability interval is defined as the range of  for which the system is composed of  stable harmonic oscillators, and is , where . The detailed stability analysis is given in the Appendix. Interestingly, the stability is independent of specific values of the 's so long as all of them are positive. Numerical computations done by the Gauss-Seidel WR algorithm verify these stability conditions, as illustrated in Figure 4.

This modified system not only introduces the mutual dependency of neighbouring components but also retains the same multitime-scale character as the original unidirectional system for a suitably chosen . Therefore, it provides a model to test the efficiency of the WR algorithm.

First, the computation is performed for  and , a case with very strong coupling among all neighbouring components. For this computation, there is a tradeoff between the multiscale efficiency and the number of iterations used. The number of iterations will depend on the coupling strength and the length of the time subinterval over which each iteration is performed (i.e., the windowing technique mentioned earlier). For this case, since the coupling is very strong, we have to reduce the subinterval to as small as  to achieve convergence with ten iterations. Here, the convergence criterion is defined by the maximum relative differences between current and previous iterates among all components, which is less than the given error constant .

Note that there always exits a small subinterval on which the WR iteration is convergent, provided that each submodel satisfies a Lipschitz condition. This can be seen from the proof of WR convergence [6]. However, it is not easy to quantify this subinterval in a general and practical way since it is context dependent. Nevertheless, in real computation this is not a significant issue, since just a few test runs will give an idea about the choice of a suitable subinterval.

However, the analysis of the computational efficiency for the unidirectional coupling above shows that the WR method is approximately 10 times faster than the standard algorithm *on a single interval*. Hence if the method is applied successively more than 10 times, the WR method is no longer efficient compared to the standard algorithm. Therefore, we further reduce the length of subinterval to . In this case, the average number of iterations required for convergence is 6 and the resulting computational efficiency is comparable with the standard algorithm. Here, we may argue that if components in a system are all coupled very strongly, then separating the system into components and performing the WR iteration would be not a good choice, and instead multirate methods [13] should be applied to the whole system. Nevertheless, it is a reasonable assumption for a biological system with an identified modular structure to exclude the existence of such strong coupling among components over long-time intervals, since the notion of modularity itself implies strong coupling within components, but weaker coupling between components.

**Figure 5:**
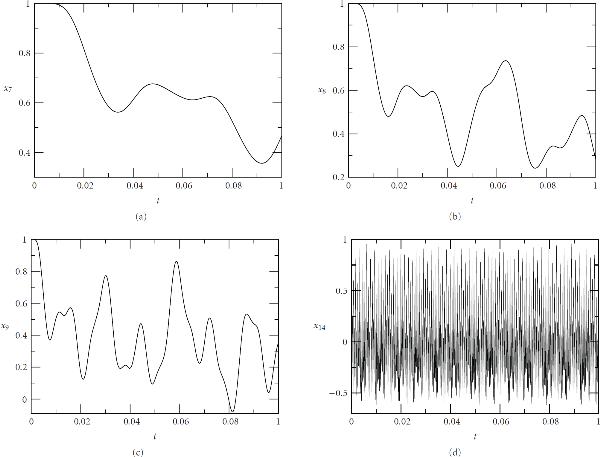
**The harmonic oscillators bidirectionally coupled with strength  for components  and strength  for components . The initial conditions: , **.

If we reduce the coupling strength  to 0.01, then a subinterval with length  will result in 5 iterations for convergence, on average. Therefore, for this weaker coupling the algorithm has a better performance than a standard algorithm. In addition, if we assume that the couplings among some of the slower components are strong, but are weak for the remaining faster components, our WR algorithm still gives a better performance than a standard algorithm. Figure 5 shows the computation for this mixed weak and strong coupling. The scenario is arranged by setting  for the first 10 components () and  for the remaining faster components (). In this situation, a discrete event-scheduled strategy applied to the iteration loops is very efficient, since it effectively senses the coupling strength and bypasses the components with very small variations.

Notice that all the comparisons above are done in terms of sequential computation, though the WR method has the obvious additional advantage of parallel computation. More precisely, the Jacobi WR method can be directly implemented in a parallel computer, that is, different processes in the computer can deal with different components simultaneously.

Because this system has purely imaginary eigenvalues, some of which have very large magnitude, Gear's method [19] based on backward differentiation is unsuitable. Instead, Prince-Dormand's embedding explicit Runge-Kutta 5(4) [20] was implemented for each component. The computations were compared with the solutions obtained with a single algorithm and agreement is achieved for all the cases discussed above (not shown).

### 4.2. Nonlinear Oscillators with Nonlinear Coupling in a Calcium Model

In [18], a model for cell calcium dynamics is presented. The main feature of the model is its ability to generate complex oscillations such as periodic bursting and chaotic bursting in response to agonist stimulation, in qualitative agreement with the complex phenomena observed in experiments. The model includes the mechanisms of feedback inhibition on the initial agonist receptor complex by calcium and activated phospholipase C (PLC), as well as receptor type-dependent self-enhanced behaviour of the activated G*α* subunit. Specifically, let  denote the concentration of active G*α* subunits,  the concentration of active PLC,  the concentration of free calcium in the cell cytosol, and  the concentration of calcium in the intracellular stores. Then this model is given by the following four nonlinear ODEs:(13)

**Figure 6:**
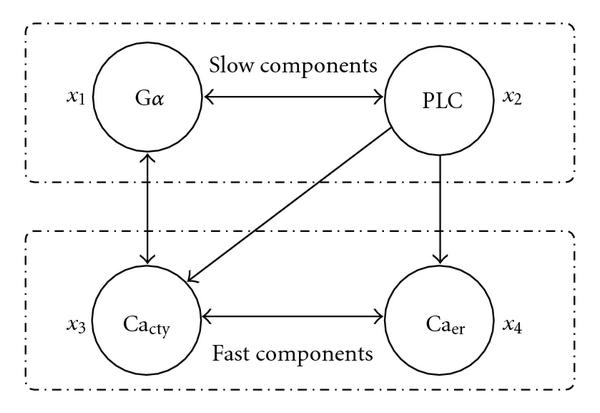
**Decomposition of Kummer's Calcium model**[18].

In the component integration approach, a natural question is how do we detect the time-scale differences among state variables so that we can define suitable components each with its own characteristic time scale? Obviously this is the key for the efficiency of multiscale algorithms. The answer comes from understanding the biology underlying the components. In this calcium model, we expect that the activity changes of G*α* and PLC in the cell membrane are relatively slow compared with the variation of calcium within the cell [21]. Therefore, we choose to partition the system into just two components, one for G*α* and PLC and the other for calcium compartments inside the cell, see Figure 6. Then we perform Gauss-Seidel WR iteration for these two coupled components. The computation confirms the supposed large difference in time scales between the two components. For example, in the computation of periodic oscillations the average adaptive step size for the first component is about 0.4 and for the second component is approximately 0.004; that is, two orders of magnitude difference. For the cases with periodic or chaotic bursting, there is still over one order of magnitude difference between the time scales of these two components.

Both Gear's method and Prince-Dormand's method, as well as their combination were applied for the computation, giving similar performance in terms of convergence, with, on average, 2-3 iterations achieving convergence to within an error constant of .

**Figure 7:**
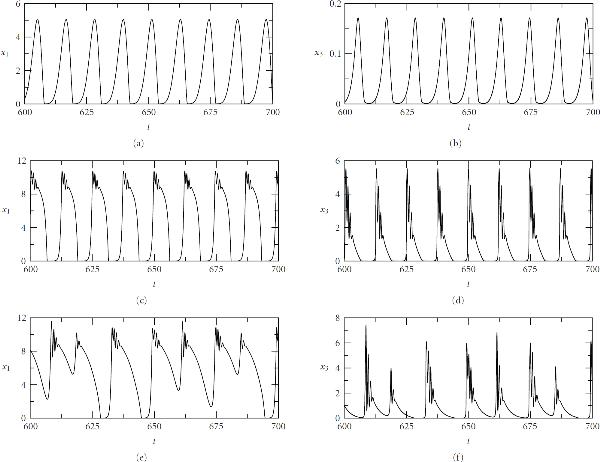
**Kummer's calcium model**[18].  is the activated G*α* subunits,  is the concentration of free calcium in the cytosol. (a) and (b): periodic oscillations with ; (c) and (d): periodic bursting with ; (e) and (f): chaotic bursting with . The initial conditions: , , , and .

This case also indicates that the WR iteration is quite robust even if we have nonlinear oscillators with nonlinear coupling between them and both periodic and chaotic bursting occur in the solutions. The results, shown in Figure 7, agree qualitatively with the computations done in the original paper [18] with a stiff ODE solver as a single integrator.

### 4.3. Stochastic/Deterministic Simulations for a Calcium Model

Höfer [17] formulated a calcium model for a hepatocyte, based on flux balances between the endoplasmic reticulum (ER) release (), the ER uptake (), the plasma membrane efflux (), the calcium influx (), and the gap-junctional flux (), resulting in the following two-dimensional system of equations(14)

where  represents the concentration of cytoplasmic free calcium, , , and  are the total areas of plasma membrane, the ER membrane, and the gap-junctional connections, respectively, and  is the effective cytosolic volume (calcium "capacity"). The free calcium content of the whole cell is , with  being the free calcium concentration in the ER. Here, we are concerned with a single cell so  is set to zero in what follows.

Stochastic simulation based on this model is done in [22]. Our purposes here are (1) to generalize Gillespie's stochastic simulation to the context of WR methodology, and (2) to show the feasibility of combining stochastic and deterministic simulation based on WR methodology. For these purposes, we reformulate the model using the variables  and , instead of  and , and also split the system into two components

Component 1:(15)

Component 2:(16)

where(17)

with  representing the concentration of inositol triphosphate (InsP3).

Based on the above model formulation, we can define stochastic processes in terms of variables  and , which are the numbers of calcium ions in the cell cytosol and the ER, respectively. Thus(18)

where  and  are the volumes of the cytosolic and ER cell compartments, respectively. Now, we can make three choices of WR simulation methods.

Method 1:

The deterministic WR. That is, we solve (16) and (17) for  and  separately and perform an iteration between the intermediate solutions to resolve the bidirectional coupling between two components.

Method 2:

The stochastic WR. Here,  and  are treated as random variables and corresponding stochastic processes are defined by infinitesimal probabilities . Thus for component 1,(19)

and for component 2(20)

Since the rate functions  and  are dependent on both  and , these two components are actually bidirectionally coupled and thus the WR method has to be used.

Method 3:

The combined deterministic and stochastic WR. Here, the state variable  for component 1 is treated as a random variable governed by the stochastic processes defined by (20), and the state variable  for component 2 is treated as a continuous variable governed by (17) and is related to  by (19). Such discrimination between components 1 and 2 is based on the fact that the concentration of calcium in the ER is much higher than that in the cytoplasm and hence is likely to be less influenced by stochastic fluctuations.

**Figure 8:**
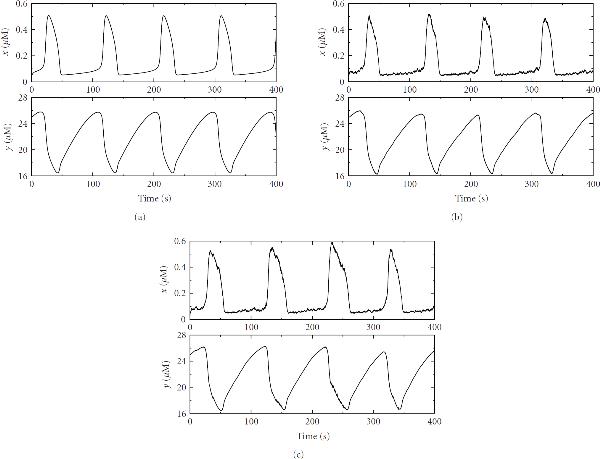
**Calcium oscillations in the WR deterministic and/or stochastic versions of Höfer's model**[17]**for a single cell**. (a) The solutions by the deterministic WR. (b) The solutions by the stochastic WR. (c) The solutions by the combined deterministic and stochastic WR. All the parameter values are taken from Höfer's original paper except for  and , which are set to be 5000  and 1000 , respectively. The Initial conditions: rest state without agonist ().

Figure 8 shows the computational output of these three methods. As expected, fluctuations appear in the stochastic simulation results, especially for the cytoplasmic-free calcium. Such a small size of fluctuations is generally observed in experimental studies. The magnitude of these fluctuations is controlled by —the volume of the cytosolic compartment of the cell. When  is large enough, fluctuations become unobservable and the solution approaches the deterministic limit, in agreement with the theory and the results obtained in [22].

### 4.4. Model for Calcium Dynamics in a Cell Plate Mediated by Gap Junctions

In [17], the single cell model was extended to consider a cell pair linked by a gap junction, and the synchronization of heterogeneous cells was studied. Using our model linking strategy, we computed a multicellular version of Höfer's cell pair.

**Figure 9:**
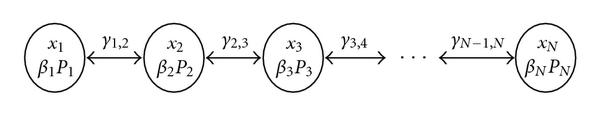
**Model for the hepatic plate**. A line of hepatic cells is bidirectionally coupled by gap junctions. Heterogeneity of the hepatocytes is controlled by the structure parameter  defined as the ratio of the effective volume of the ER to the effective cytosolic volume. The concentration of InsP3 is denoted by .  denotes the concentration of cytoplasmic-free calcium in cell .  represents the coupling coefficient as defined by the scaled gap junction permeability. The model is based on Höfer's calcium oscillation model in hepatocytes [17].

The model (see Figure 9) can be interpreted as representing a plate of hepatocytes spanning from the portal space to the hepatic venule in the liver, in agreement with the known anatomy. The liver plate is a sheet of cells approximately 10 cells high and 15–25 cells long [23]. For the purposes of computation, each cell in a vertical stack was taken to be equivalent and the spread of signal computed for a line of cells with the signal travelling in one dimension along the plate. The communication of cells in the plate is through gap junctions, and the cell-surface receptor density—the binding of hormone ligands to which activates the calcium pathway—is allowed to vary along the plate. The multicellular extension of Höfer's model is summarised as follows:(21)

Here, each cell acts as a (nonlinear) calcium oscillator linearly coupled with its neighbours. The strength of the coupling is , representing the scaled gap junction permeability. The heterogeneity amongst cells comes from different sources: the variation of InsP3 levels (represented by  in the model) due to the density differences of receptors on different cell membranes, and the varying capacity of calcium stores between cells represented by the structure parameters , defined as the ratio of the effective volume of the ER to the effective cytosolic volume. In the computations, InsP3 levels () among the cells are set up with a constant gradient, reflecting an experimentally observed density gradient of cell surface receptors between periportal and perivenous zones [24]. The structure parameters  are treated as constants or random factors varying between 0.1 and 0.2 (these values are chosen from Höfer's estimation [17]).

**Figure 10:**
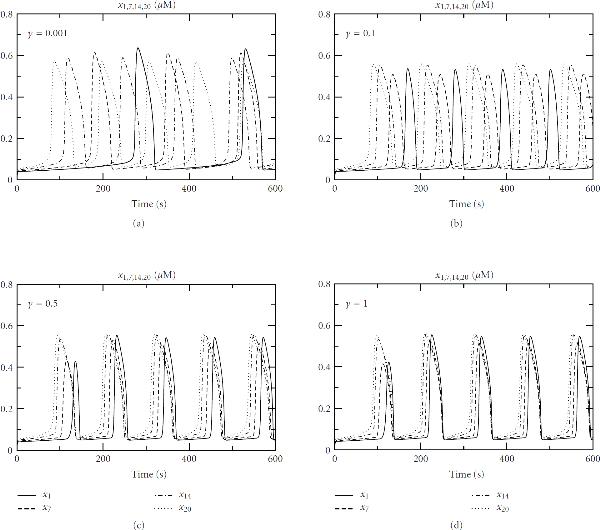
**Synchronization of calcium oscillations in a plate of hepatocytes linked by gap junctions: , , , , , , and . Initial conditions: rest state without agonist ()**.

**Figure 11:**
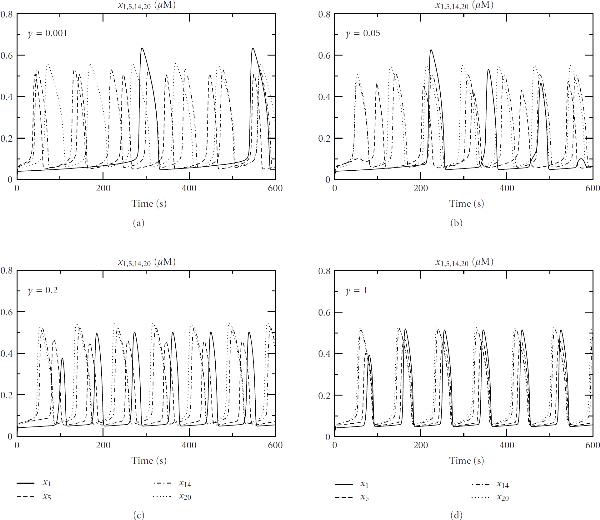
**Synchronization of calcium oscillations in a plate of hepatocytes linked by gap junctions: , , , and  are randomly distributed between 0.1 and 0.2**. In this particular computation, , , , and . Initial conditions: rest state without agonist ().

Calcium oscillations are inherent to individual cells and the frequency and the shape of oscillations are determined by many factors, such as cell type, ligand and receptor densities, and so forth. In this particular model for hepatocytes, the oscillation frequency is governed by InsP3 levels and structural parameters. Therefore, for weak gap-junctional coupling (small values of ), the calcium in each cell oscillates at its own inherent frequency, as indicated in Figures 10 and 11. However, when the permeability of gap junctions  is increased, the computations show that the oscillations for individual cells become synchronized towards the frequency for the cell with the highest InsP3 level (Figures 10 and 11). This implies that the cell with the highest InsP3 level will direct the calcium waves in the liver lobule, in agreement with Höfer's analysis of a cell pair and also with experimental studies [24].

When the Gauss-Seidel WR is applied to solve these bidirectionally coupled components, the convergence rate depends on the coupling strength . For a weak coupling, say , or 3, iterations are sufficient to achieve convergence, but for an increasing  the number of iterations increases.

## 5. Discussion and Conclusions

The main goal of systems biology and computational physiology is to formulate predictive models of integrated, functional biological systems which exploit the massive increase in low-level (usually molecular) detailed understanding and data that has accumulated over the past few decades. In parallel with biological developments, the history of mathematical and computational modelling of biological systems has largely concentrated on discrete, well-described, single-scale, and isolatable subsystems. Taking the agenda of systems biology forward will require the integration of a number of these small-scale models of (comparatively) well-understood mechanisms into larger models in order to investigate the effects of dynamical interactions between the model components (e.g., for discrete multicellular systems such as organs). Further, several large and complicated models may need to be linked together to derive a biologically sensible model at a still larger scale (e.g., for whole-organism physiological systems).

A major challenge, therefore, is to take existing and new mathematical models of biological subsystems and integrate them together into new and larger systems. This is a difficult task to achieve for a number of reasons. First is the difficulty of delineating the appropriate biological modular structures, and identifying (or constructing) models of each such module. Second, the nature of biological interfaces between modules, and their representation as couplings amongst submodels must be formulated. A wide variation in the mathematical representation and software implementation of submodels often makes it impractical (or, at least, inefficient) to integrate submodels by simply reformulating them within a common computational paradigm. More fundamentally, it is impractical to construct a general reformulation framework to include all such potential variation. Because of this, any such special case reformulation will have limited generalizability. Third is designing an integration methodology that allows for efficient computation. Thus from both a practical point of view and a system level perspective, a general methodology that provides a means to capture the global solutions over time and space independently of the solution techniques related to individual submodels or components is required.

In this study, we propose waveform relaxation (WR) as a general methodology to integrate biological models to create large models of functional systems. The WR numerical method for parallel integration of systems of coupled ODEs is particularly useful because it allows the realisation of an integrated model without reformulating the whole problem, regardless of what formalisms were originally employed for each of the component models. We have demonstrated by case studies the multitime scale efficiency for bidirectionally coupled components, the convergence robustness, the flexibility, and the capability of tackling model heterogeneity of the WR method.

In principle, the WR strategy can be applied to a model system at any level of organisation, from the level of elementary chemical reactions to that of interactions in ecosystems. However, the efficiency of the method will depend on both the network structure and the dynamics of the network, as we demonstrate in Section 4.1. This closely relates to the difficulty associated with defining the concept of modularity [25]. The main purpose of this paper is to provide a practical means—the WR method—for integrating model systems. Pragmatic, but informed, judgement concerning these issues will always be required.

## Appendix

### Stability of The Bidirectionally Coupled Harmonic Oscillator System

Let . Then we can rewrite the system defined by (13) as(A.1)

where the Jacobian matrix **D** is  block triagonal:(A.2)

with(A.3)

We are required to find conditions under which **D** has only complex eigenvalues, and hence that the system is decomposable into a system of stable harmonic oscillators. Thus if  is the characteristic polynomial of **D**, we consider conditions under which  for all real .

Consider the LU-decomposition of the tridiagonal block matrix , where(A.4)

Then(A.5)

It now follows easily from (A.3) and (A.5) that(A.6)(A.7)

From (A.6), we have  for , where we set . It follows easily by induction from (A.7) that  for all real  and all .

Set  and(A.8)

for . Then  implies that(A.9)

It follows that  for all real  and  if and only if  for each .

Clearly,  for all real . Assume inductively that  for all real  and . Then  implies that(A.10)

Thus setting(A.11)

it follows by induction that  for all real , and all  if and only if(A.12)

We now obtain the following.

Theorem A.1.

For , , and real , the system (A.1) consists of  stable harmonic oscillators if and only if , where(A.13)

Thus  is monotonically decreasing in , and .

Proof.

It remains to identify the set . From (A.7), we have(A.14)

Substituting from (A.9) gives(A.15)

for , and hence(A.16)

The solution of the difference equation (A.16) with initial condition  is(A.17)

where  are the roots of the quadratic . That is, . Clearly, these are real and distinct for  and complex for .

Setting , for  transforms (A.17) to(A.18)

and we conclude that , for all  and .

For , set  with . Then (A.17) gives(A.19)

where . Thus  if and only if  for positive integer . This gives a finite set of possible values , for , and hence a corresponding finite set of real roots of :(A.20)

Clearly,  is the smallest of these roots. Also,  is decreasing in , and hence , for . It, therefore, follows that , for  and all , which gives (A.13) and completes the proof of the theorem.
